# Oral Health Status and Treatment Needs among Pregnant Women of Raichur District, India: A Population Based Cross-Sectional Study

**DOI:** 10.1155/2016/9860387

**Published:** 2016-05-12

**Authors:** Ritu Gupta, Arun Kumar Acharya

**Affiliations:** ^1^Department of Public Health Dentistry, DJ College of Dental Sciences and Research, Modinagar, Uttar Pradesh 201204, India; ^2^Department of Public Health Dentistry, Navodaya Dental College and Hospital, Raichur, Karnataka 584102, India

## Abstract

*Background and Objectives*. Pregnancy can be a risk factor for dental diseases as oral tissues are liable to changes due to hormonal variations. The aim of the study was to assess the oral health status and treatment needs among pregnant women of Raichur district, Karnataka, India.* Methods*. Cross-sectional data was collected from 300 primigravidae from all the 5 taluks of Raichur district visiting the respective community health centre at taluk headquarters. A specially designed questionnaire was used to assess the demographic variables and oral hygiene practices. A clinical examination was done according to WHO (World Health Organization) criteria 1997 and recorded using WHO Oral Health Assessment Form.* Results*. The mean age of the pregnant women in the study was 21.8 (2.12) years. The prevalence of caries and periodontal diseases was 62.7% and 95%, respectively. The mean DT, MT, FT, and DMFT were 2.06 (2.5), 0.03 (0.17), 0.04 (0.27), and 2.13 (2.54), respectively. The mean OHI-S was 2.87 (1.27). Chi-square test showed that CPI scores increased with the trimester of pregnancy.* Conclusion*. The present study demonstrates poor oral hygiene and high prevalence of periodontal diseases, as well as a large proportion of unmet dental treatment needs among pregnant women of Raichur district, India.

## 1. Introduction

The healthy future of society depends on the health of the children of today and their mothers, who are guardians of that future [1]. Since the old wives' tale of “the loss of tooth for every pregnancy,” oral health during pregnancy has long been a focus of interest [2]. The 9-month period of pregnancy in a woman's life is defined not only by the development of her unborn child but also by the adaptive changes that her body undergoes to support the pregnancy. Pregnancy is a dynamic physiological state evidenced by several transient changes. These changes may develop into various physical signs and symptoms that can affect the individual's health, perceptions, and interactions with others in her environment [3].

In a woman's life, the major physiological and hormonal changes occur during pregnancy. The most significant hormonal changes in pregnancy are the increased levels of estrogen and progesterone [4]. Estradiol levels in plasma are reported to increase up to 30 times higher than those found during a reproductive cycle. Estrogen regulates cellular proliferation, differentiation, and keratinisation. But progesterone affects the permeability of the microvasculature and also changes the production of collagen [5]. It is these hormonal alterations during pregnancy that tend to increase the incidence of dental diseases like gingivitis and may even contribute to low salivary pH, thus in turn leading to increased incidence of dental caries [6].

Gingival changes usually occur in association with poor oral hygiene and local irritants such as calculus, plaque, and food debris. The bacterial flora of dental plaque is noted to be the main etiological factor in the causation of gingival and periodontal diseases [4, 7]. However, the hormonal and vascular changes that accompany pregnancy often exaggerate the inflammatory response to the local irritants. The gingiva without preexisting inflammation remains unaffected [4].

The prevalence of periodontal disease ranges from 30% [8] to 100% [9] in some reports. Furthermore, high prevalence of dental caries of 74% [10] and 99.9% [11] has been reported among pregnant women.

Pregnancy gingivitis is a nonspecific, vascularizing, and proliferative inflammation with large amounts of infiltrated inflammatory cells. The onset of pregnancy gingivitis coincides with the selective growth of periodontal pathogens, such as* Prevotella intermedia*, in subgingival plaque from 3rd-4th month of pregnancy [12]. Increased proportion of* Prevotella intermedia* (previously* Bacteroides intermedius*), concomitant with an increase in gingivitis and elevated serum levels of estrogen and progesterone, have been shown during pregnancy [13].

During pregnancy, gingival inflammation increases significantly from the first to the third trimester [9], with a maximum increase in the second trimester [6, 12, 14] and a decrease at the end of 3 months postpartum [12]. Multiple studies have associated periodontal disease with premature birth and low birth weight of infants [15–19].

Description of the epidemiological situation is the cornerstone of a successful preparation and implementation of the preventive program. It is important to know the oral health status of pregnant women in order to recommend effective preventive measures.

Dentistry can be vital in improving prenatal outcome and maternal or foetal dental health through screening, referral, and education of pregnant women. It is important to understand that establishing a healthy oral examination is the most important objective in planning dental care for pregnant women.

Literature is sparse on the study of oral health status and treatment needs of pregnant women experiencing their first pregnancy. Hence, the present study was conducted to assess the oral health status and treatment needs of primigravidae of Raichur district to rule out the influence of previous pregnancies on the result obtained.

## 2. Material and Methods

A descriptive cross-sectional study was conducted to assess oral health status and treatment needs among pregnant women of Raichur district, Karnataka, India.

Raichur district is an administrative district in Karnataka, India. It is located in the northeastern part of the state [20]. It has a population of 19,28,812, according to 2011 census [21]. Raichur district for administrative purpose has been divided into five taluks: Raichur, Manvi, Devadurga, Sindhanur, and Lingasugur. The population of Raichur is primarily rural with 75% rural population and just 25% living in urban areas [20].

The present study was conducted at the community health centre of each of the five taluk headquarters where the pregnant women have access to preventive, diagnostic, and curative services and most of the pregnant women are referred to taluka hospital for scanning and other types of care from various subcentres, primary health centres, and other community health centres. Therefore, these community health centres were chosen to collect the baseline data regarding the oral health status and treatment needs among pregnant women of Raichur district. All the pregnant women examined were registered under the Department of Health and Family Welfare, Raichur district, and were “Tai” card holders under which they could avail benefits of various government schemes.

### 2.1. Source of Data

A total sample of 300 primigravidae visiting the community health centres of 5 taluks of Raichur district were included in the study.

#### 2.1.1. Sample Size Determination and Sampling Procedure

The sample size for the present study was calculated based on the data obtained from the pilot study conducted in community health centres of 5 taluks of Raichur district. The prevalence of periodontal disease among primigravidae was found to be 75% in the study. For the present study, the sample size was determined at 95% confidence interval.

Sample size was calculated using the following formula:

(1) $$\begin{array}{c} n=\frac{z^{2}\times p\times q}{m^{2}}, \end{array}$$

where “*n*” is the number of subjects required, *z* is confidence level at 95% (standard value of 1.96), *p* = 75% (prevalence of periodontal disease) = 0.75, *q* = 1 − *p* = 25% = 0.25, *m* = margin of error at 5% (standard value of 0.05), *n* = 1.96^2^ × 0.75 × 0.25/0.05 × 0.05, and *n* = 288.12 ≈ 288 subjects rounded off to 300.

The total sample of 300 women was proportionately selected in each taluk based on the total population of each taluk.

### 2.2. Inclusion Criteria

Primigravidae who gave written consent were included in the study. The pregnancies were confirmed through a clinical examination of the pregnant women by the qualified doctor.

### 2.3. Exclusion Criteria

Pregnant women giving history of medications, current use of systemic corticosteroids, congenital heart disease, existing hypertension and diabetes before the pregnancy, and history of epilepsy, asthma, and chronic renal disease and pregnant women with multiple deliveries or whose infants were stillborn were excluded from the study.

The systemic illnesses were ruled out after medical history was taken by the qualified doctor.

### 2.4. Collection of Data

The study protocol was approved by the Institutional Ethical Committee and Review board, Raichur. The permission and consent was obtained from the District Health Officer, Raichur district, to conduct the study in all the five taluks.

Voluntary informed written permission was obtained from the study participants after explanation of the nature of the study. The script was presented both in English and Kannada for easy understanding and convenience of the study participants. Patients unable to read the consent form were helped by communicating the details in the presence of or through the accompanying person if required.

All the data collected was recorded in a pro forma by a trained assistant. The data was collected over a period of 6 months from January to June 2014. Recording of data was done in the antenatal unit of the Department of Obstetrics and Gynaecology of all the five community health centres.

### 2.5. Questionnaire

A specially prepared structured questionnaire was interviewer-administered to the pregnant women to know the demographic variables and oral hygiene practices. All the questions were explained in the local language and the answers were recorded by the examiner.

An assessment of per capita income classified according to Agarwal's modification of B. G. Prasad scale [22] for the socioeconomic status was used.

### 2.6. Clinical Examination

Pregnant women were examined for oral health status, treatment needs, and oral hygiene. Oral health status and treatment needs were assessed using the WHO Oral Health Assessment Form (1997) [23]. Oral hygiene was assessed using Oral Hygiene Index-Simplified and was recorded according to the criteria given by Greene and Vermillion in 1964 [24].

### 2.7. Training and Calibration

Two-day training sessions for standardization and calibration of the data collection methods were organized in the Department of Public Health Dentistry. The training session consisted of a revaluation of the criteria outlined, followed by an examination of adult patients based on simulation of field technique for reliability. Oral health status was assessed using WHO Oral Health Assessment Form (1997). Intraexaminer reliability was assessed through Cohen's kappa which was 0.97.

## 3. Statistical Analysis

Data was analyzed using SPSS v16.0 software package. Descriptive statistics such as mean, standard deviation, and percentage were used. Association was evaluated using chi-square. Any *p* value less than 0.05 was considered significant.

## 4. Results

The present study was conducted on primigravidae aged 18–26 years with a mean age of 21.8 (2.12) years.

### 4.1. Demographic Characteristics

Table 1 shows the sociodemographic characteristics of the pregnant women. The study was conducted in all the five taluks of Raichur district, and the sample was proportionately collected based on the population of each taluk. More than half of the pregnant women, 152 (50.7%), were poor. Sample was taken almost equally from all the three trimesters of pregnancy. A total of 188 (62.7%) used toothbrush with toothpaste to clean their teeth, while nearly one-third, 34.3%, used finger and charcoal to clean their teeth.

### 4.2. Extraoral Examination and Temporomandibular Joint Assessment

The pregnant women examined had normal extraoral appearance, and none of the pregnant women examined had any symptoms of temporomandibular joint (TMJ) disorder, whereas only 3 (1.0%) pregnant women showed signs of TMJ disorder, that is, reduced jaw mobility, with no pregnant women in the study showing signs of clicking and tenderness on palpation of TMJ.

### 4.3. Oral Mucosa Examination

On intraoral examination of the oral mucosa, 281 (93.7%) of the pregnant women had healthy mucosa, 01 (0.3%) had leukoplakia, 01 (0.3%) had ulceration, 10 (3.4%) had oral submucous fibrosis, 01 (0.3%) had fissured tongue, and 06 (2.0%) had pyogenic granuloma (pregnancy epulis) and all the pregnant women with pyogenic granuloma were in their third trimester of pregnancy.

### 4.4. Periodontal Condition

Periodontal condition was assessed using CPI (community periodontal index); only 5.0% of the pregnant women had healthy periodontium. The mean number of sextants per person with healthy periodontium was 0.8, while with calculus it was 3.7.

### 4.5. Periodontal Loss of Attachment

The present study showed no periodontal loss of attachment in 273 (91.0%) of the pregnant women (Table 2).

### 4.6. Dentition Status and Treatment Needs

The prevalence of dental caries among pregnant women was 62.7%.

Tables 3 and 4 show mean number of teeth/person according to dentition status and treatment needs code.

The mean DMFT/person in the present study was 2.13 (2.54), mean DT/person was 2.06 ± 2.5, mean MT/person was 0.03 ± 0.17, and FT/person was 0.04 (0.27).

### 4.7. Prosthetic Status and Prosthetic Needs

In the present study, only 3 (1%) of the pregnant women had prosthesis in the upper jaw that is bridge. None of the pregnant women had prosthesis in the lower jaw.

Of the 300 pregnant women examined, 288 (96.0%) pregnant women did not require any prosthesis in the upper jaw, 09 (3.0%) required one-unit prosthesis, 02 (0.7%) required multiunit prosthesis, and 01 (0.3%) required a combination of one- and/or multiunit prosthesis in the upper jaw.

Similarly, 272 (90.7%) pregnant women did not require a prosthesis in the lower jaw, 19 (6.3%) required one-unit prosthesis, 04 (1.3%) required multiunit prosthesis in the lower jaw, and 05 (1.7%) required a combination of one- and/or multiunit prosthesis in the lower jaw.

### 4.8. Need for Immediate Care and Referral

There was a need for immediate care and referral for 10 (3.3%) of the subjects who presented with oral submucous fibrosis, 01 (3.3%) pregnant woman who presented with leukoplakia, and 56 (18.7%) pregnant women who presented with either pain or infection due to dental caries.

### 4.9. Oral Hygiene Status (Oral Hygiene Index-Simplified)

The Oral Hygiene Index-Simplified was assessed for all the 300 pregnant women; 46 (15.3%) had good oral hygiene, 122 (40.7%) had fair oral hygiene, and 132 (44.0%) had poor oral hygiene, with mean debris-index simplified score being 1.59 (0.58), mean calculus-index simplified score being 1.28 (0.77), and the mean Oral Hygiene Index-Simplified score per person being 2.87 (1.29).

### 4.10. Gestational Age and Community Periodontal Index, Periodontal Loss of Attachment, and Oral Hygiene Index-Simplified

Statistically significant association between gestational age and periodontal condition of the pregnant women was seen (*p* < 0.05). Periodontal loss of attachment measuring 4-5 mm was seen to be more prevalent among the pregnant women in their third trimester (13.9%). No significant association between gestational age and loss of periodontal attachment and oral hygiene was observed in the present study (Table 5).

## 5. Discussion

Pregnancy constitutes a special physiological state characterized by a series of temporary adaptive changes in the body structure as a result of increased production of reproductive hormones that include estrogen, progesterone, gonadotropins, and relaxin. The oral cavity is also affected by such endocrine actions and may present both transient and irreversible changes as well as modifications that are considered pathological [25].

In the present study, the mean age of pregnant women was low, 21.8 (2.12) years, as according to a report, in Raichur district, 89.6% of women aged 20–24 years are married [26]. The sample was collected from pregnant women at various stages of pregnancy to facilitate comparison of oral health status at these stages. Pregnant women with no systemic illness or conditions considered to be risk factors for adverse pregnancy outcomes which could potentially confound the study variables were included and primigravidae were only included in order to rule out the influence of previous pregnancies on the results obtained.

Socioeconomic status is one of the risk factors for poor health and oral health outcomes. Persons of lower socioeconomic status suffer disproportionately more from nearly all diseases than people of higher socioeconomic status [27]. In the present study, 66% of the women were of lower socioeconomic status followed by lower middle class and very low fraction of the pregnant women studied were from high socioeconomic status. These findings are in contrast to the study done by Murthy et al. on primigravidae in Belgaum, India [28], where majority (85.0%) of the pregnant women belonged to upper class and no pregnant women were reported from poor and below poverty line classes when classified according to Agarwal's modification of B. G. Prasad scale. This could be due to difference in the sampling frame of the two studies with the present study being done only in community health centres.

In the present study, nearly one-third (34.3%) of the pregnant women used finger and charcoal to clean their teeth; these findings are in contrast with the studies by Al-Turck in Riyadh, Saudi Arabia [29], Gürsoy et al. in Finland [30], and Christensen et al. in Denmark [31], where all the pregnant women examined used toothbrush. The poor oral hygiene measures of pregnant women in the present study could be due to lack of awareness among these pregnant women of simple oral health preventive measures (oral hygiene) as majority of pregnant women belonged to economically weak background who paid less attention to oral health.

The prevalence of oral mucosal lesions in the present study was 6.3%, which is comparable to study done on pregnant women by Annan and Nuamah in Accra, Ghana (4%) [32]. The prevalence of pregnancy epulis, which is a form of pyogenic granuloma, was 2%, similar to the study on pregnant women in Accra, Ghana (3%) [32]. The relation between these vascular lesions and pregnancy is possibly due to high levels of corticosteroids, though this has not yet been proven directly [32].

The prevalence of periodontal diseases was high in the present study with only 5.0% of the pregnant women having healthy periodontium. The high prevalence (95%) of periodontal disease in the present study is comparable to studies done on pregnant women by Jago et al. in Brisbane, Australia (97%) [33], Miyazaki et al. in Japan (95%) [34], and Tonello et al. in Lucas do Verde, MT, Brazil (83.0%) [35], but the findings are higher than the prevalence of periodontal diseases among pregnant women of the study done by Wandera et al. in Mbale district, Uganda (67.3%) [36], and Arafat in Baltimore (76.7%) [37]. The increased prevalence of periodontitis could be due to the poorer oral hygiene status of the pregnant women that may have aggravated the influence of hormones on the periodontium. This could also be due to most pregnant women being illiterate and belonging to poor or below poverty line classes. Also, it could be due to lack of knowledge about the competent practices of good oral hygiene measures as in the present study.

The present study showed periodontal loss of attachment measuring 4-5 mm in only 8.7% of the study subjects; this cannot be compared with those of other studies, since although gingival inflammatory changes have been reported loss of attachment has not been measured in most of the studies. In a follow-up study done by Tilakaratne et al. in Sri Lanka [12] and Taani et al. in Irbid Governorate, Jordan [38], the clinical loss of attachment remained unchanged in all the three trimesters among the pregnant women. This could probably be due to elevated hormone levels, characteristic of pregnancy not affecting the periodontal attachment. The most likely explanation for this result is that elevated levels of these hormones during the 9-month period is insufficient to cause significant breakdown, despite their reported effects on the epithelial barrier, vasculature, and connective tissue matrix [12].

The population of present study depicted worsening of the periodontal condition with the progression of pregnancy that was found to be statistically significant (*p* < 0.05). An increase in the severity of periodontal changes with the progression of pregnancy was also observed in studies conducted by Yalcin et al. in Istanbul, Turkey [39], Taani et al. in Irbid Governorate, Jordan [38], and Lieff et al. in North Carolina [40].

The observed prevalence of dental caries in the current study is similar to findings of Murthy et al. in Belgaum (60.4%), India [28]. The mean DMFT of the current study population is similar to that of the study done by Pentapati et al. in Manipal, India (3.08) [41] but is comparatively lower than the caries experience of pregnant women of Brisbane (15.8) [33] and Kaunas, Lithuania (12.1) [11]. The low dental caries experience in the present study might be due to less exposure to refined carbohydrates and sugars as the majority belonged to low socioeconomic status and also due to racial, ethnic, and economic disparity in sugar consumption between developed and developing countries.

The analysis of component parts of the mean DMFT of our study demonstrates that the mean number of decayed teeth was in excess in comparison to missing teeth and filled teeth which is comparable to the study done by Karunachandra et al. in rural Sri Lanka [42] and Pentapati et al. in Manipal, India [41], but is in contrast with a study on pregnant women conducted by Christensen et al. in Brisbane [31], where the filled teeth component (8.6) was in excess of the missing (4.5) and decayed (2.7) component. This depicts lower utilization of dental services and large portion of unmet needs in our study population.

The pregnant women of Raichur district were found to have a very low prosthetic status, which is in contrast with the study done in Brisbane [33]. This may be due to low caries experience and the age group of pregnant women in our study being 18–26, whereas the mean DMFT in the study done in Brisbane was 15.8 with the pregnant women examined being in the age range of 16–42 years.

Oral hygiene-simplified index scores were found to increase progressively with the increase in trimester of pregnancy, which could probably be due to the less importance that is given to oral health with progression of pregnancy. This finding is supported by the observations of the studies on pregnant women by Arafat in Baltimore [37] and Samant et al. in Chandigarh [14].

It was observed in the present study that 95% of the pregnant women required treatment, in the form of either restorative care, pulp care, extraction, prosthesis, oral prophylaxis, or advanced periodontal treatment. Restorative care in the form of one surface filling and two or more surface fillings was required by 49.3% and 16.7% of the pregnant women, respectively, with a mean of 1.4 teeth per person requiring one surface filling and 0.23 teeth per person requiring two or more surface filings (15.3%); that is, 0.19 teeth per person needed pulp care and restoration and only 10% and 0.2 teeth per person needed extraction of unrestorable teeth (root stumps or grossly decayed tooth). The prosthetic need in the present study was also very low with only 4% requiring partial dentures in the upper jaw and 9.3% in the lower jaw and none having the need for complete dentures. In another study done on pregnant women by Jago et al. in Brisbane [33], 70% of the pregnant women required restorative care, where on an average 2.7 teeth per person were in need of restoration, and 10% needed extraction of at least one tooth, with 0.2 teeth per person needing extraction, 5% required some form of partial denture, and no women required new full dentures.

The study shows very low utilization of dental services by pregnant women with an attitude of negligence towards their oral health. Oral health is given little importance in contrast to the impending motherhood.

## 6. Conclusion

There is a need to improve the oral health knowledge and oral health care habits of pregnant women of Raichur district by making oral health an integral part of antenatal and primary health care in order to prevent oral diseases and encourage them to have regular dental care to prevent further disease.

Prenatal and perinatal oral health along with infant oral health is one of the foundations upon which preventive education and dental care must be built to enhance the opportunity for a child to have a lifetime free from preventable oral diseases. Pregnancy offers an opportunity to educate women regarding oral health by providing a “teachable moment” in self-care with future children [43].

## Figures and Tables

**Table 1:** Sociodemographic characteristics of 300 pregnant women from Raichur district, India.

	Number	Percentage
*Age group (years)*		
18–20	98	32.7
21–23	128	42.7
24–26	74	24.6
*Geographic location*		
Raichur, rural	40	13.3
Raichur, urban	38	12.7
Manvi	60	20.0
Sindhanur	65	21.7
Devadurga	37	12.3
Lingasugur	60	20.0
*Socioeconomic status*		
Upper high	03	1.0
High	11	3.7
Upper middle	21	7.0
Lower middle	67	22.3
Poor	152	50.7
Below poverty line	46	15.3
*Gestational age*		
First trimester	97	32.3
Second trimester	102	34.0
Third trimester	101	33.7
*Oral hygiene practices*		
Toothbrush and toothpaste	188	62.7
Finger and toothpaste	05	1.7
Finger and toothpowder	03	1.0
Finger and charcoal	103	34.3
Only finger	01	0.3

**Table 2:** Distribution of pregnant women according to loss of attachment.

Loss of attachment	Number	Percentage
0 mm	273	91.0
4-5 mm	26	8.7
6–8 mm	00	0.0
9–11 mm	01	0.3
12 mm or more	00	0.0
Excluded sextant	00	0.0
Not recorded	00	0.0

*Total*	*300*	*100.0*

**Table 3:** Mean number of permanent teeth per person according to dentition status code.

Dentition status	Mean number of teeth per person (SD)
Sound	28.84 (3.18)
Decayed	2.05 (2.48)
Filled, with decay	0.17 (0.15)
Filled, no decay	0.04 (0.27)
Missing as a result of caries	0.02 (0.17)
Missing for any other reason	0.01 (0.13)
Bridge abutment, special crown, or veneer implant	0.05 (0.57)
Unerupted tooth (crown)/unexposed root	0.96 (1.67)
Trauma	0.01 (0.08)

SD: standard deviation.

**Table 4:** Mean number of teeth per person according to treatment required.

Type of treatment	Mean number of teeth per person (SD)
Preventive care	0.05 (0.29)
Fissure sealant	0.14 (0.56)
One surface filling	1.44 (2.08)
Two or more surface fillings	0.23 (0.58)
Crown	0.04 (0.23)
Pulp care and restoration	0.19 (0.47)
Extraction	0.2 (0.7)

*Total*	*2.29 (2.58)*

SD: standard deviation.

**Table 5:** Association between gestational age, periodontal condition, loss of attachment, and oral hygiene status in pregnant women.

Gestational age	Number (%) of persons coded						
	Periodontal condition						
	Healthy	Bleeding	Calculus	Pocket	Pocket	Total	
				4-5 mm	≥6 mm		
First trimester	08 (8.2)	10 (10.3)	64 (66.0)	10 (10.3)	05 (5.2)	97 (100.0)	^*∗*^ *p* < 0.05
Second trimester	04 (3.9)	05 (4.9)	55 (53.9)	34 (33.4)	04 (3.9)	102 (100.0)	
Third trimester	03 (3.0)	07 (6.9)	47 (46.5)	33 (32.7)	11 (10.9)	101 (100.0)	

*Total*	*15 (5.0)*	*22 (7.3)*	*166 (55.3)*	*77 (25.7)*	*20 (6.7)*	*300 (100.0)*	

	Loss of attachment						
	0–3 mm	4-5 mm		9–11 mm		Total	

First trimester	89 (91.8)	08 (8.2)		00 (0.0)		97 (100.0)	*p* > 0.05
Second trimester	97 (95.1)	04 (3.9)		01 (1.0)		102 (100.0)	
Third trimester	87 (86.1)	14 (13.9)		00 (0.0)		101 (100.0)	

*Total*	*273 (91.0)*	*26 (8.7)*		*01 (0.3)*		*300 (100.0)*	

	Oral hygiene status						
	Good	Fair		Poor		Total	

First trimester	19 (19.6)	41 (42.3)		37 (38.1)		97 (100.0)	*p* > 0.05
Second trimester	14 (13.7)	40 (39.2)		48 (47.1)		102 (100.0)	
Third trimester	13 (12.9)	41 (40.6)		47 (46.5)		101 (100.0)	

*Total*	*46 (15.3)*	*122 (40.7)*		*132 (44.0)*		*300 (100.0)*	

^*∗*^Statistically significant at *p* < 0.05.

## References

[1] World Health Organization (2005). The World Health Report 2005: make every mother and child count. *Mother and Children Matter—So Does Their Health*.

[2] Gaffield M. L., Colley Gilbert B. J., Malvitz D. M., Romaguera R. (2001). Oral health during pregnancy: an analysis of information collected by the pregnancy risk assessment monitoring system. *Journal of the American Dental Association*.

[3] Dellinger T. M., Livingston H. M. (2006). Pregnancy: physiologic changes and considerations for dental patients. *Dental Clinics of North America*.

[4] Laine M. A. (2002). Effect of pregnancy on periodontal and dental health. *Acta Odontologica Scandinavica*.

[5] Yaghobi S., Haghighati F. (2011). Evaluation of oral health status and treatment needs for periodontal treatment in pregnant women. *DJH*.

[6] Tadakamadla S. K., Agarwal P., Jain P. (2007). Dental Status and its Socio-demographic influences among pregnant women attending a maternity hospital in India. *Revista de Clínica e Pesquisa Odontológica*.

[7] Ojanotko-Harri A. O., Harri M. P., Hurttia H. M., Sewón L. A. (1991). Altered tissue metabolism of progesterone in pregnancy gingivitis and granuloma. *Journal of Clinical Periodontology*.

[8] Hasson E. (1960). Pregnancy gingivitis. *Harefuah*.

[9] Löe H., Silness J. (1963). Periodontal disease in pregnancy I. Prevalence and severity. *Acta Odontologica Scandinavica*.

[10] Rakchanok N., Amporn D., Yoshida Y., Harun-Or-Rashid M., Sakamoto J. (2010). Dental caries and gingivitis among pregnant and non-pregnant women in Chiang Mai, Thailand. *Nagoya Journal of Medical Science*.

[11] Vasiliauskiene I. (2003). Oral health status of pregnant women. *Stomatologija*.

[12] Tilakaratne A., Soory M., Ranasinghe A. W., Corea S. M. X., Ekanayake S. L., De Silva M. (2000). Periodontal disease status during pregnancy and 3 months post-partum, in a rural population of Sri-Lankan women. *Journal of Clinical Periodontology*.

[13] Kornman K. S., Loesche W. J. (1980). The subgingival microbial flora during pregnancy. *Journal of Periodontal Research*.

[14] Samant A., Malik C. P., Chabra S. K., Devi P. K. (1976). Gingivitis and periodontal disease in pregnancy. *Journal of Periodontology*.

[15] Offenbacher S., Lieff S., Boggess K. A. (2001). Maternal periodontitis and prematurity. Part I: obstetric outcome of prematurity and growth restriction. *Annals of Periodontology*.

[16] López N. J., Smith P. C., Gutierrez J. (2002). Periodontal therapy may reduce the risk of preterm low birth weight in women with periodontal disease: a randomized controlled trial. *Journal of Periodontology*.

[17] Mokeem S. A., Molla G. N., Al-Jewair T. S. (2004). The prevalence and relationship between periodontal disease and pre-term low birth weight infants at King Khalid University Hospital in Riyadh, Saudi Arabia. *Journal of Contemporary Dental Practice*.

[18] Marin C., Segura-Egea J. J., Martínez-Sahuquillo Á., Bullón P. (2005). Correlation between infant birth weight and mother's periodontal status. *Journal of Clinical Periodontology*.

[19] Radnai M., Gorzó I., Urbán E., Eller J., Novák T., Pál A. (2006). Possible association between mother's periodontal status and preterm delivery. *Journal of Clinical Periodontology*.

[20] Raichur K. (2011). *District Health and Family Welfare Department. Maternal, Newborn and Child Health in Raichur, a Situation Analysis*.

[21] http://www.raichur.nic.in/DSO/RaG(2012-13).pdf.

[22] Agarwal A. K. (2008). Social classification: the need to update in the present scenario. *Indian Journal of Community Medicine*.

[23] World Health Organization (1997). *Oral Health Surveys-Basic Methods*.

[24] Greene J. C., Vermillion J. R. (1964). The simplified oral hygiene index. *Journal of the American Dental Association*.

[25] Díaz-Guzmán L. M., Castellanos-Suárez J. L. (2004). Lesions of the oral mucosa and periodontal disease behavior in pregnant patients. *Medicina Oral, Patologia Oral y Cirugia Bucal*.

[27] Hughes D., Simpson L. The role of social change in preventing low birth weight. http://futureofchildren.org/publications/journals/article/index.xml?journalid=60&articleid=374&sectionid=2511.

[28] Murthy S., Mubashir A., Kodkany B. S., Mallapur M. D. (2012). Pregnancy periodontitis and low birth weight: a cohort study in rural Belgaum, India. *Global Journal of Medicine and Public Health*.

[29] Al-Turck K. M. A. (2005). Self-reported dental care and dietary habits of Saudi pregnant women in prenatal clinic in Riyadh. *Pakistan Oral and Dental Journal*.

[30] Gürsoy M., Pajukanta R., Sorsa T., Könönen E. (2008). Clinical changes in periodontium during pregnancy and post-partum. *Journal of Clinical Periodontology*.

[31] Christensen L. B., Jeppe-Jensen D., Petersen P. E. (2003). Self-reported gingival conditions and self-care in the oral health of Danish women during pregnancy. *Journal of Clinical Periodontology*.

[32] Annan B. D. R. T., Nuamah K. (2005). Oral pathologies seen in pregnant and non-pregnant women. *Ghana Medical Journal*.

[33] Jago J. D., Chapman P. J., Aitken J. F., McEniery T. M. (1984). Dental status of pregnant women attending a Brisbane maternity hospital. *Community Dentistry and Oral Epidemiology*.

[34] Miyazaki H., Yamashita Y., Shirahama R. (1991). Periodontal condition of pregnant women assessed by CPITN. *Journal of Clinical Periodontology*.

[35] Tonello A. S., Zuchieri M. A. B. O., Pardi V. (2007). Assessment of oral health status of pregnant women participating in a family health program in the city of Lucas do Rio Verde—MT—Brazil. *Brazilian Journal of Oral Sciences*.

[36] Wandera M., Engebretsen I. M. S., Okullo I., Tumwine J. K., Åstrøm A. N. (2009). Socio-demographic factors related to periodontal status and tooth loss of pregnant women in Mbale District, Uganda. *BMC Oral Health*.

[37] Arafat A. H. (1974). Periodontal status during pregnancy. *Journal of Periodontology*.

[38] Taani D. Q., Habashneh R., Hammad M. M., Batieha A. (2003). The periodontal status of pregnant women and its relationship with socio-demographic and clinical variables. *Journal of Oral Rehabilitation*.

[39] Yalcin F., Eskinazi E., Soydinc M. (2002). The effect of sociocultural status on periodontal conditions in pregnancy. *Journal of Periodontology*.

[40] Lieff S., Boggess K. A., Murtha A. P. (2004). The oral conditions and pregnancy study: periodontal status of a cohort of pregnant women. *Journal of Periodontology*.

[41] Pentapati K. C., Acharya S., Bhat M., Rao S. K., Singh S. (2013). Knowledge of dental decay and associated factors among pregnant women: a study from rural India. *Oral Health & Preventive Dentistry*.

[42] Karunachandra N. N., Perera I. R., Fernando G. (2012). Oral health status during pregnancy: rural–urban comparisons of oral disease burden among antenatal women in Sri Lanka. *Rural and Remote Health*.

[43] Guidelines on Perinatal Oral Health Care (2011). *Clinical Guidelines*.

